# The Association Between Craniofacial Morphological Parameters and the Severity of Obstructive Sleep Apnea: A Multivariate Analysis Using the Apnea–Hypopnea Index and Nocturnal Oxygen Desaturation

**DOI:** 10.3390/healthcare13080913

**Published:** 2025-04-16

**Authors:** Zhili Dong, Jinmei Wu, Liping Wu, Hong Hong

**Affiliations:** 1 Hospital of Stomatology, Guangdong Provincial Clinical Research Center of Oral Diseases, Guanghua School of Stomatology, Sun Yat-sen University, Guangzhou 510055, China; dongzhli@mail2.sysu.edu.cn; 2 Longyan First Affiliated Hospital of Fujian Medical University, Longyan 364000, China; m13665072114@163.com

**Keywords:** apnea–hypopnea index, cephalometric parameters, lateral cephalogram, lowest nocturnal oxygen saturation, obstructive sleep apnea

## Abstract

**Background**: Obstructive sleep apnea (OSA) is characterized by repetitive complete or partial closure of the upper airway during sleep, which is a potentially life-threatening disorder. A cephalogram is a simple and effective examination to predict the risk of OSA in orthodontic clinical practice. This study aims to analyze the relationship between craniofacial characteristics and the severity of OSA using polysomnography and cephalogram data. Gender differences in these parameters are also investigated. **Methods**: This study included 112 patients who underwent a complete clinical examination, standard polysomnography study, and cephalometric analysis to diagnose obstructive sleep apnea. This study divided the participants into male and female groups to study the correlation between cephalometric parameters and the severity of OSA. The analysis involved 39 cephalometric parameters. The severity of obstructive sleep apnea was evaluated by the apnea–hypopnea index (AHI) and the lowest nocturnal oxygen saturation (LSaO_2_). **Results**: The final assessment included 112 adult participants (male/female = 67:45, mean age: 28.4 ± 7.29 years, mean male age: 28.8 ± 7.62 years, mean female age: 27.8 ± 6.79 years). Multivariate analysis revealed that the mandibular position, incisor inclination, facial height, and maxillary first molar position were strongly associated with OSA severity. Gender-specific differences in cephalometric predictors were identified, with distinct parameters correlating with the AHI and LSaO_2_ in males and females. Notably, the LSaO_2_ demonstrated stronger associations with craniofacial morphology in females than males. **Conclusions**: Cephalometric analysis can be effective in assessing the risk and severity of OSA based on the correlation between cephalometric parameters and the AHI/LSaO_2_. There is a clear difference between the cephalometric parameters associated with OSA severity in male and female individuals. This gender-dependent pattern may assist the personalized diagnosis and management of OSA in clinical practice.

## 1. Introduction

Obstructive sleep apnea (OSA) refers to a prevalent disorder characterized by repetitive episodes of upper airway collapse during sleep that disrupts normal sleep patterns and ventilation, resulting in symptoms such as snoring, witnessed apnea, and excessive daytime sleepiness [1]. As a very common but frequently undiagnosed disease, recent estimates suggest that OSA affects 9% of middle-aged men and 4% of adult women, with approximately half experiencing moderate-to-severe forms of the condition [2,3]. Risk factors for OSA include obesity, male sex, age, menopause, hypothyroidism, goiter (independent of thyroid function), fluid retention, adenotonsillar hypertrophy, and smoking [4,5]. In pediatric patients, OSA has been associated with neurocognitive deficits, difficulties in learning, behavioral issues, stunted growth, and impaired cardiac function [6,7]. The elderly population is particularly vulnerable to this global health issue since untreated OSA is linked to a number of potential negative health consequences, including hypertension, diabetes mellitus, stroke, atherosclerosis, and an elevated risk of cardiovascular mortality [8,9]. Therefore, early screening and diagnosis are essential for reducing the negative impact of OSA on general health. While standard polysomnography (both in-lab and home-based PSG) remains the gold standard for OSA diagnosis, it does not inherently provide detailed morphological evaluation of the craniofacial region. This limitation underscores the need for adjunctive tools to identify anatomical risk factors of OSA, especially for those who may receive orthodontic or orthognathic treatment [10,11]. Anatomical parameters including airway width and length, hyoid bone position, tongue volume, and other craniofacial structures are determinate factors in the pathogenesis of certain OSA cases, hence complementary diagnostic tools are needed for a comprehensive evaluation of these factors [12].

Although advanced imaging techniques such as cone-beam computed tomography (CBCT) and magnetic resonance imaging (MRI) have been widely adopted for evaluating the morphological details of orofacial structures, cephalometry persists as a valuable diagnostic tool for assessing skeletal and soft-tissue relationships. Its continued relevance in clinical practice stems from three key advantages: the cost-effectiveness compared to higher-resolution modalities, significantly reduced radiation exposure, and a technical simplicity that enables efficient implementation in routine screening protocols [13,14]. Certain craniofacial skeletal abnormalities have been linked to an increased risk of OSA, such as maxillary and mandibular retrognathia, an increased mandibular plane angle, and an inferiorly positioned hyoid bone, which makes lateral cephalogram an effective tool for the early screening of OSA [15,16]. The current diagnostic and severity grading criteria for OSA are primarily based on the apnea–hypopnea index (AHI), which represents the number of apnea and/or hypopnea events occurring per hour during sleep, regardless of the related nocturnal oxygen desaturation. The severity of OSA can be classified as mild (6 ≤ AHI ≤ 15), moderate (16 ≤ AHI ≤ 29), or severe (AHI ≥ 30) [17]. Similarly, most of the cephalometric studies have investigated the correlation between craniofacial morphological characteristics and the severity of OSA based solely on the AHI [15]. However, substantial evidence suggests that the AHI alone does not fully reflect the clinical symptoms and prognosis of patients with OSA. Mediano et al. reported that patients with similar AHIs may exhibit varying levels of daytime sleepiness, sleep latency, and nocturnal oxygenation [18]. Asano et al. found that although some patients share a similar mean AHI, the severity of hypoxia and incidence of cardiovascular events were significantly different [19]. Wang et al. suggested that an oxygen saturation below 90% during the total sleep time was independently associated with the risk of hypertension in patients with severe OSA, even after adjustment for traditional risk factors such as the AHI and body mass index (BMI) [20]. Building upon existing evidence, systematic evaluation of cephalometric correlations with nocturnal oxygen saturation in patients with OSA represents a critical step in identifying anatomical predictors of hypoxia risk and informing clinical management strategies. This study aims to investigate cephalometric predictors of OSA severity by analyzing craniofacial morphology through lateral cephalometric radiography. Two polysomnographic parameters—the AHI and LSaO₂—are utilized as primary severity indicators, enabling the identification of skeletal and soft-tissue biomarkers associated with OSA severity.

## 2. Materials and Methods

### 2.1. Study Design and Samples

The study was designed as a retrospective study. The study population consisted of patients referred to the Department of Orthodontics at the Hospital of Stomatology, Sun Yat-sen University, between January 2018 and July 2024. This study was approved by the Medical Ethics Committee of the Hospital of Stomatology, Sun Yat-sen University (Approval No.KQEC-2024-99-01). The inclusion criteria included the following: age over 18 years; a diagnosis for OSA confirmed by polysomnography (PSG); and the availability of a digital lateral cephalogram taken with the same cephalostat with the patient in an upright position, with a natural head posture and centric occlusion [21]. Patients were excluded if they had undergone continuous positive airway pressure (CPAP) treatment, had craniofacial or growth abnormalities, or had a history of orthodontic treatment or craniofacial trauma and surgery. Based on the inclusion/exclusion criteria, a total of 112 patients were included in this observational prospective study. All data used in this study were obtained from medical record review.

### 2.2. PSG

All participants underwent standard overnight polysomnographic monitoring, which included EEGs (C3/A2 and C4/A1, measured using surface electrodes), electrooculograms (measured using surface electrodes), submental electromyograms (measured by surface electrodes), nasal airflow (measured using a nasal cannula with a pressure transducer), oral airflow (measured with a thermistor), chest wall and abdominal movements (recorded by inductance plethysmography), electrocardiography, and pulse oximetry. Respiratory events were classified according to the American Academy of Sleep Medicine Criteria 2012 (version 2.0) [22]. The AHI was defined as apneas plus hypopneas per hour during sleep. Consistent with the 2012 criteria, hypopneas were defined as ≥30% reductions in airflow accompanied by either a ≥3% oxygen desaturation or an arousal. The AHI and lowest oxygen saturation (LSaO_2_) were recorded for further analysis in this study.

### 2.3. Cephalometric Analysis

**Figure 1:**
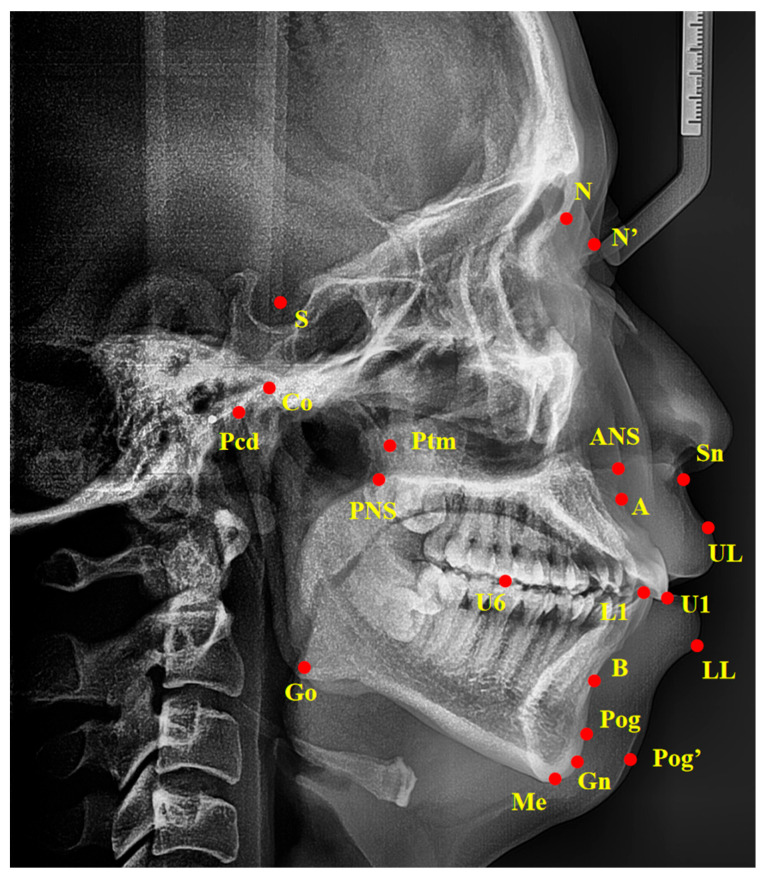
Landmarks of cephalometric analysis. Ba, basion; S, sella point; N, nasion; ANS, anterior nasal spine; PNS, posterior nasal spine; A, the deepest point in the concavity of the anterior maxilla between the anterior nasal spine and the alveolar crest; B, the deepest point in the concavity of the anterior mandible between the alveolar crest and pogonion; Pcd, the most posterosuperior point of the condylar head; Go, gonion; Gn, gnathion; Me, menton; Ptm, pterygomaxillary fissure point; Pog, pogonion; U1, upper incisors; L1, lower incisors; U6, the mesial buccal cusp of upper maxillary first molar; UL, upper lip; LL, lower lip; N’, nasion of soft tissue; Pog’, pogonion of soft tissue; Sn, subnasale.

Standardized lateral cephalograms were taken with the same cephalostat at the Hospital of Stomatology, Sun Yat-sen University, with the patient in an upright position, with a natural head posture and centric occlusion. Cephalometric tracings were performed by an examiner blind to the PSG reports and clinical examination results, using the Digident software, version 2.10 (Boltzmann Zhibei Technology Co., Ltd., Chengdu, China) to calculate all angular and linear measurements [23]. All landmarks were automatically digitized and manually adjusted on each radiograph. Thirty-nine variables of linear and angular measurements were calculated from fourteen landmarks digitized on each radiograph as described in Figure 1 and Table 1. To evaluate the error of the method, 20 cephalograms were selected randomly and duplicate determinations were performed 4 weeks later by the same examiner. An intra-class correlation (ICC) coefficient was calculated to evaluate the intra-operator reliability. The values of all calculated intra-class correlation coefficients were greater than 0.95, showing repeated agreement with regard to all measurements.

### 2.4. Statistical Analysis

SPSS 25.0 (SPSS Inc., Chicago, IL, USA) was used to perform the statistical analysis. Descriptive data were presented as the mean ± standard deviation (SD), median with interquartile range (IQR), or number (percentage), as appropriate. Whether the data were normally distributed was examined using the Shapiro–Wilk test. The independent *t*-test (for normally distributed variables) and non-parametric Mann–Whitney U test (for non-normally distributed variables) were used to compare the differences in the demographic characteristics between male and female subjects. Correlation coefficients were measured through the Pearson correlation analysis if the variables followed a normal distribution, while those that were not normally distributed were examined through the Spearman rank correlation analysis. Based on the univariate analyses, the correlation between the cephalometric variables and the severity of OSA was determined by multiple regression analysis. *p* ≤ 0.05 was considered statistically significant.

Sample size calculation was performed based on the study by Yu et al. [24]. In order to detect a correlation of 0.45 (−0.45) using a two-sided hypothesis test with a significance level of 0.05 and a power of 0.80, a number of 36 subjects would be needed for each group.

## 3. Results

### 3.1. Demographic Data

A total of 112 adult participants (M/F = 67:45, mean age: 28.4 ± 7.29 years, age range: 18–52 years, mean male age: 28.8 ± 7.62 years, mean female age: 27.8 ± 6.79 years) were included in this study. The detailed demographic data of the patients are demonstrated in Table 2. The AHI value and the LSaO_2_ levels, along with 13 out of 39 cephalometric parameters were significantly different in the different gender groups. We further divided the participants into male and female groups to investigate whether gender differences affect the correlation between craniofacial parameters and OSA severity.

### 3.2. Correlation Analysis Between Craniofacial Cephalometric Parameters and the Severity of OSA in All Subjects Regardless of Gender

In this study, we investigated the relations between cephalometric parameters and OSA severity indicators (the AHI and LSaO_2_) in all subjects regardless of gender. Regarding the AHI, SNA, SNB, PP-FH, U1-L1, U1-SN, L1-NB (°), and L1-Apo were the parameters with statistically significant correlations with the AHI. SNA, SNB, U1-SN, L1-NB (°), and L1-Apo were negatively correlated with the AHI, while PP-FH and U1-L1 were positively correlated with the AHI. Interestingly, none of the cephalometric parameters included in this study demonstrated statistically significant correlation with the LSaO_2_ in the whole sample (Table 3).

### 3.3. Correlation Analysis Between Craniofacial Cephalometric Parameters and the Severity of OSA in Male and Female Individuals

Based on the results above, we investigated whether there was a gender-dependent pattern of the cephalometric parameters associated with the AHI and LSaO_2_. In male individuals, only U1-L1 and U1-SN were the parameters with a statistically significant correlation with the AHI. Meanwhile, SNB, ANS-Me, L1-NB (°), and L1-Apo demonstrated a statistically significant correlation with the AHI in the female groups (Table 4). Regarding the LSaO_2_, U6-PP and N’-SN-Pog’ were identified as parameters with a statistically significant correlation in the male group. In the female group, SNB, ANB, NBa-PtGn, and U6-Ptm were statistically significant parameters (Table 5). As expected, all of the correlation coefficients in the male and female groups showed a similar trend to the whole sample regardless of gender (Table 3, Table 4 and Table 5).

### 3.4. Multiple Regression Analysis Between Cephalometric Parameters and OSA Severity Indicators in the Male and Female Groups

Based on the results of the correlation analysis above, we selected the parameters that showed a strong correlation with the AHI and LSaO_2_ in both gender groups to perform a multiple regression analysis.

The multiple regression model was designed to predict the severity of OSA based on the cephalometric parameters. The AHI and LSaO_2_ were used as dependent variables to study the correlation between other independent variables. The VIF values between one and five of the variables indicated moderate multicollinearity between the variables and the AHI/LSaO_2_. Multivariate regression analysis demonstrated explanatory power for all models, with R^2^ values of 0.124 (male AHI), 0.218 (female AHI), 0.202 (male LSaO₂), and 0.469 (female LsaO_2_). All models showed statistical significance in the F-test (*p* < 0.05) (Table 6 and Table 7).

## 4. Discussion

While the AHI has played a pivotal role in OSA classification, its limitations as a standalone diagnostic marker have become increasingly evident [25]. Emerging evidence highlights the need to integrate multidimensional metrics, such as hypoxia burden (e.g., T90, LSaO_2_), blood biomarkers, and genetic predictors, to refine OSA risk stratification [26]. Notably, nocturnal hypoxia severity demonstrates stronger associations with OSA-related comorbidities such as hypertension, type 2 diabetes, and systemic inflammation compared to the AHI alone [20,27,28]. Furthermore, pretreatment oxygen saturation levels correlate closely with therapeutic efficacy in improving oxygenation [29]. These findings advocate for combining the AHI with the LSaO_2_ and clinical assessments to optimize OSA diagnosis and personalized management.

As an effective tool to identify the risk of OSA, cephalometric analysis was often adopted to investigate the occurrence of OSA based on the AHI [30,31]. Its clinical utility lies in identifying craniofacial predictors of hypoxia susceptibility rather than replacing diagnostic sleep studies. Recent advances in AI-driven cephalometric analysis demonstrate remarkable reproducibility and diagnostic concordance with expert clinicians, potentially overcoming traditional limitations of manual tracing [32,33]. Such automated systems could enable standardized OSA risk stratification, even in primary care settings [34]. According to The American Association of Orthodontists (AAO) white paper on OSA and orthodontics, the strength of the relationship between craniofacial morphologies and the development of OSA is not well established [35]. It is also unclear as to whether the anatomic features of the craniofacial region can reflect the severity of nocturnal hypoxia. In this study, we explored the relations between morphological characteristics on a cephalogram and the severity of OSA based on both the AHI and LSaO_2_. As expected, there are several cephalometric parameters that showed a statistically significant correlation with the AHI and LSaO_2_, and the parameters were different in male and female groups.

In the correlation analysis for the whole sample, seven parameters correlated significantly with the AHI: five negatively (SNA, SNB, U1-SN, L1-NB°, L1-Apo) and two positively (PP-FH, U1-L1), while none of the cephalometric parameters showed a statistically significant correlation with the LSaO_2_. However, after dividing the subjects into different gender groups, we observed obvious changes in the parameters. Only U1-L1 and U1-SN were the parameters with a statistically significant correlation with the AHI in the male group, indicating that a more labial-inclined upper incisor position in relation to the lower incisors, and the SN line is related to a higher severity of OSA. In the female group, SNB, ANS-Me, L1-NB (°), and L1-Apo were all negatively correlated with the AHI (Table 4), suggesting that a more retruded mandible, a lower facial height, and less labial inclination of the lower incisors are associated with a higher severity of OSA.

Regarding the LSaO_2_, none of the parameters that significantly correlated with the AHI are associated with the LSaO_2_, and only U6-PP and N’-SN-Pog’ were identified as parameters with a statistically significant correlation in the male group. Based on the correlation coefficient value, a longer distance from the U6 to PP line and a larger N’-SN-Pog’ angle indicate a higher severity of OSA and risk of nocturnal hypoxia. In the female group, SNB, ANB, NBa-PtGn, and U6-Ptm were statistically significant parameters, with ANB being the only parameter that was negatively correlated with the LSaO_2_, suggesting that a more retruded and clockwise-rotated mandible, and a shorter distance from U6 to Ptm are associated with a higher risk of nocturnal hypoxia (Table 5). Interestingly, it has been observed that in patients with OSA without cardiovascular disease at baseline but who are followed up for a median duration of 78 months, the burden of hypoxia rather than the AHI predicted cardiovascular events and mortality after adjusting for confounding factors; notably stronger associations were observed in patients under 65 years and in women [36]. Our study also revealed that more cephalometric parameters exhibited associations with the LSaO_2_ in females compared to males; furthermore, the correlation levels between these parameters and the LSaO_2_ were also higher among females than males. These results implicate potential correlations between craniofacial morphology and the severity of OSA complications, along with a gender-dependent pattern.

Based on the correlation analysis mentioned above, we performed a multiple regression analysis to examine the viability of using cephalometric parameters to predict the values of the AHI and LSaO_2_. All of the regression models are statistically effective according to the F examination, but the level of correlation is relatively low. Notably, the level of correlation is significantly higher in the LSaO_2_ models than the AHI models, with almost twice the R^2^ value in the male and female groups, respectively. The results suggest that the cephalometric analysis is effective in assessing the LSaO_2_, and it is more reliable in predicting the severity of OSA based on the LSaO_2_ rather than the AHI. Regarding the gender difference, previous studies have shown that the prevalence and severity of OSA are described to be much higher in men, which could be related to sex hormones, changes in body fat distribution, neck circumference. and central ventilatory control [37,38]. It is quite interesting to find out that a gender-dependent pattern also applies to the cephalometric parameters related to the OSA severity in adult individuals. Further studies should investigate whether the difference in cephalometric parameters in male and female individuals contribute to the different clinical manifestations of OSA. In addition, the study population exhibited a younger mean age compared to prior OSA prevalence studies, which predominantly involved middle-aged and elderly cohorts [39]. This discrepancy may reflect sampling bias inherent to orthodontic clinics, where younger individuals disproportionately seek treatment. Therefore, caution should be exercised when extrapolating these findings to broader populations of patients with OSA.

An abnormality in craniofacial morphology is a crucial predisposing factor in the pathogenesis of OSA, which is often evaluated by cephalometric analysis by orthodontists [40,41]. However, according to the American Academy of Sleep Medicine’s Clinical Practice Guideline, the physician specializing in sleep medicine is considered to be the primary healthcare provider with the highest qualification for diagnosing and treating patients with OSA [42,43]. Therefore, it appears that orthodontists fall outside of the scope of OSA diagnosis and management. In fact, since many patients with OSA seek orthodontic treatment for malocclusion, orthodontists can easily conduct early screening assessments for OSA [44]. By using lateral cephalograms, orthodontists can evaluate the patient’s craniofacial morphological features and assess the risk of OSA. In addition, when developing orthodontic treatment plans, orthodontists have a responsibility to consider how their interventions may impact the prognosis of OSA. While maxillomandibular advancement (MMA) surgery has proven to be the more successful surgical intervention for OSA, apart from tracheostomy, its association with orthognathic surgery and higher cost often discourage patient selection [45]. On the other hand, mandibular advancement devices (MADs) demonstrate efficacy for certain patients but are typically considered as a last-resort due to potential adverse effects. Other orthodontic treatments for OSA such as maxillary expansion either lack sufficient reliability or require long-term clinical follow-up [46,47]. Therefore, based on the results of this study and previous research, we suggest that orthodontists should pay more attention to cephalometric parameters related to the AHI and LSaO_2_. It is worth noting that cephalometric analysis only offers orthodontists and oral–maxillofacial surgeons a practical framework for the early identification of high-risk individuals since it is rooted in morphological assessment. If the cephalometric analysis indicates a higher risk of OSA, it is recommended that the patient consults a sleep specialist for further advice.

This investigation has several limitations. First, the sample consisted exclusively of Chinese patients with a relatively small cohort size. As a retrospective study design, our analysis was confined to pre-existing lateral cephalograms and polysomnography data, inherently limiting the inclusion of a control group or additional oximetric parameters (e.g., T90, oxygen desaturation index) that better quantify the hypoxemia burden. In addition, the potential impact of artifactually low LSaO_2_ values due to transient technical factors (e.g., oximeter displacement) could not be definitively ruled out in this retrospective analysis. Finally, several confounding variables, including body mass index and neck circumference, were not systematically controlled for due to incomplete medical records.

## 5. Conclusions

Several cephalometric parameters are related to the severity of OSA, as indicated by the AHI and LSaO_2_. Some of these parameters exhibit gender-specific differences. There is a significant difference between the parameters associated with the AHI and those associated with the LSaO_2_. Our analysis suggest that cephalometric parameters may exhibit gender-dependent associations with OSA severity, particularly when integrating the LSaO_2_ as a hypoxia-specific metric. While these findings highlight potential anatomical dimorphism in OSA pathophysiology, further validation in diverse cohorts is required before gender-specific cephalometric criteria can be implemented. Orthodontists should interpret these trends cautiously, prioritizing individualized risk assessment alongside polysomnographic evaluation.

## Figures and Tables

**Table 1:** Description of the cephalometric measurements.

Measurements	Description
**Angular measurements (°)**	
SNA	Angle between point S and A at N
SNB	Angle between point S and B at N
ANB	Angle between point A and B at N
PP-FH	Angle between PP plane and FH plane
PP-GoGn	Angle between PP plane and line Go–Gn
OP-SN	Angle between occlusal plane and SN plane
MP-SN	Angle between mandibular plane and SN plane
FH-MP	Angle between FH plane and mandibular plane
SGn-FH	Angle between line S–Gn and FH plane
NBa-PtGn	Angle between line N–Ba and line Pt–Gn
U1-L1	Angle between the long axis of upper incisors and lower incisors
U1-SN	Angle between the long axis of upper incisors and SN plane
U1-NA	Angle between the long axis of upper incisors and line N–A
L1-NB	Angle between the long axis of lower incisors and line N–B
L1-FH	Angle between the long axis of lower incisors and FH plane
Z-Angle	Angle between line Z-line and FH plane
FH-N’Pog’	Angle between FH plane and line N’Pog’
N’-Sn-Pog’	Angle between point N’ and Pog’ at Sn
**Linear measurements (mm)**	
Ptm-A	Distance between Ptm and A
Ptm-S	Distance between Ptm and S
Go-Pog	Distance between Go and Pog
Go-Co	Distance between Go and Co
Pcd-S	Distance between Pcd and S
N-ANS	Distance between N and ANS
ANS-Me	Distance between ANS and Me
S-Go	Distance between S and Go
U1-NA	Distance between U1 and line N–A
L1-NB	Distance between L1 and line N–B
U1-APo	Distance between U1 and line A–Pog
L1-APo	Distance between L1 and line A–Pog
U6-Ptm	Distance between U6 and Ptm
U1-PP	Distance between U1 and PP plane
U6-PP	Distance between U6 and PP plane
L1-MP	Distance between L1 and mandibular plane
L6-MP	Distance between L6 and mandibular plane
UL-EP	Distance between UL and EP plane
LL-EP	Distance between LL and EP plane

Note: SN plane: the line connecting S and N; FH plane: Frankfort plane; MP: mandibular plane; PP plane: the line connecting ANS and PNS; Z-line: The line that connects the most prominent point on soft-tissue chin with the most prominent point on either upper or lower lip, depending on which lip was more protruded; EP plane: The line drawn from the tip of the nose to the tip of the chin.

**Table 2:** Demographic, polysomnographic, and cephalometric parameters.

	Male	Female	Total	*p*-Value (Male vs. Female)
Number	67	45	112	-
Age	28.8 ± 7.6	27.8 ± 6.8	28.4 ± 7.3	0.469
**Polysomnography parameters**				
AHI	11.8 (7.20, 19.0)	7.40 (5.20, 11,1)	10.0 (6.00, 17.9)	0.001 **
LSaO_2_ (%)	86.0 (80.0, 90.0)	89.0 (85.0, 92.0)	87.0 (82.0, 91.0)	0.019 *
**Cephalometric parameters**				
SNA	80.9 ± 3.7	82.0 ± 3.6	81.3 ± 3.7	0.095
SNB	74.1 ± 5.0	73.6 ± 4.7	73.9 ± 4.9	0.534
ANB	6.7 (5.1, 7.9)	8.5 ± 3.1	7.2 (5.8, 8.8)	<0.001 ***
Ptm-A	44.6 ± 3.4	43.8 ± 3.9	44.3 ± 3.6	0.303
Ptm-S	17.6 ± 3.6	18.5 ± 2.7	18.0 ± 3.3	0.185
PP-FH	3.2 ± 3.0	2.6 ± 3.3	3.0 ± 3.1	0.272
PP-GoGn	28.8 ± 8.0	32.3 ± 8.9	30.2 ± 8.5	0.028 *
OP-SN	21.4 (17.2, 25.6)	22.6 (18.1, 28.0)	21.7 (18.0, 26.3)	0.320
Go-Pog	67.1 ± 6.4	64.8 ± 8.0	66.2 ± 7.2	0.092
Go-Co	55.2 ± 6.1	51.1 ± 6.5	54.4 (50.5, 57.5)	0.001 **
Pcd-S	16.3 ± 3.2	14.6 ± 2.6	15.6 ± 3.1	0.003
MP-SN	40.8 ± 10.0	44.6 ± 9.3	42.3 ± 9.8	0.044 *
FH-MP	33.6 ± 9.8	36.8 ± 9.7	34.9 ± 9.9	0.098
SGn-FH	68.3 ± 5.5	69.2 ± 5.2	68.6 ± 5.3	0.388
NBa-PtGn	82.4 ± 7.4	79.3 ± 7.7	81.2 ± 7.6	0.037 *
N-ANS	54.7 ± 3.8	52.0 (50.9, 54.4)	53.4 (51.2, 56.1)	0.001 **
ANS-Me	64.8 ± 7.3	63.2 ± 8.5	64.2 ± 7.8	0.297
S-Go	74.9 ± 7.7	69.9 ± 7.2	72.9 ± 7.8	<0.001 ***
S-Go/N-Me	62.8 ± 5.5	60.3 ± 4.4	61.8 ± 5.2	0.013 *
ANS-Me/N-Me	54.1 ± 2.4	54.3 ± 3.3	54.2 ± 2.8	0.723
U1-L1	121 ± 11.8	117 (110, 124)	120 ± 11.7	0.214
U1-SN	99.3 ± 9.0	98.3 ± 8.8	98.9 ± 8.9	0.562
U1-NA (mm)	4.4 ± 3.7	4.4 ± 3.8	4.4 ± 3.7	0.941
U1-NA (°)	18.5 ± 8.8	16.7 ± 7.4	17.8 ± 8.2	0.267
L1-NB (mm)	9.0 ± 3.2	10.5 ± 3.6	9.62 ± 3.4	0.022 *
L1-NB (°)	34.3 ± 7.6	36.5 ± 6.5	35.2 ± 7.2	0.114
L1-FH	47.0 ± 10.4	44.9 ± 7.6	46.1 ± 9.4	0.243
U1-APo	10.4 ± 3.7	12.6 ± 4.2	11.3 ± 4.1	0.004 **
L1-APo	4.9 ± 3.8	6.0 ± 3.7	5.3 ± 3.8	0.117
U6-Ptm	20.5 ± 4.2	19.0 ± 3.8	19.9 ± 4.1	0.057
U1-PP	30.8 ± 3.1	31.3 ± 3.7	31.0 ± 3.3	0.452
U6-PP	23.8 ± 3.3	23.5 ± 3.1	23.7 ± 3.2	0.568
L1-MP	43.0 ± 4.4	43.2 ± 5.0	43.1 ± 4.6	0.757
L6-MP	34.1 ± 3.6	33.0 ± 4.0	33.6 ± 3.8	0.118
UL-EP	2.96 ± 3.2	5.06 ± 3.0	3.8 ± 3.3	<0.001 ***
LL-EP	3.57 ± 3.7	6.10 ± 3.4	4.6 ± 3.8	<0.001 ***
Z-Angle	54.3 ± 13.5	48.5 ± 11.0	52.0 ± 12.8	0.018 *
FH-N’Pog’	84.3 ± 6.2	83.6 ± 5.2	84.0 ± 5.8	0.549
N’-SN-Pog’	154.0 ± 7.5	152.0 ± 6.7	153.0 ± 7.2	0.094

* indicates *p* < 0.05; ** indicates *p* < 0.01; *** indicates *p* < 0.001.

**Table 3:** Correlation analysis between cephalometric parameters and AHI/LSaO_2_ in all subjects.

	AHI		LSaO_2_	
	r	*p*	r	*p*
SNA	−0.195	0.039	0.132	0.166
SNB	−0.248	0.008 **	0.131	0.167
ANB	0.107	0.261	−0.016	0.865
Ptm-A	0.046	0.633	0.015	0.878
Ptm-S	−0.081	0.394	−0.087	0.363
PP-FH	0.205	0.030 *	−0.041	0.665
PP-GoGn	−0.133	0.162	0.04	0.674
OP-SN	0.003	0.977	0.075	0.431
Go-Pog	−0.148	0.121	0.038	0.689
Go-Co	0.044	0.648	−0.122	0.201
Pcd-S	0.169	0.075	−0.039	0.681
MP-SN	0.006	0.952	−0.015	0.878
FH-MP	−0.012	0.899	0.016	0.866
SGn-FH	0.101	0.291	−0.016	0.863
NBa-PtGn	−0.1	0.294	0.1	0.292
N-ANS	0.134	0.158	−0.121	0.202
ANS-Me	−0.096	0.315	−0.039	0.685
S-Go	0.055	0.567	−0.106	0.265
S-Go/N-Me	0.083	0.387	−0.051	0.594
ANS-Me/N-Me	−0.18	0.058	0.015	0.877
U1-L1	0.209	0.027 *	−0.114	0.230
U1-SN	−0.211	0.025 *	0.102	0.283
U1-NA (mm)	−0.123	0.195	0.043	0.654
U1-NA (°)	−0.084	0.381	0.017	0.860
L1-NB (mm)	−0.12	0.209	0.052	0.583
L1-NB (°)	−0.258	0.006 **	0.137	0.150
L1-FH	0.071	0.455	−0.062	0.517
U1-APo	−0.056	0.560	0.031	0.747
L1-APo	−0.239	0.011 *	0.078	0.415
U6-Ptm	−0.073	0.447	−0.026	0.748
U1-PP	−0.069	0.467	−0.014	0.885
U6-PP	0.014	0.885	−0.104	0.273
L1-MP	−0.01	0.919	−0.078	0.413
L6-MP	0.053	0.582	−0.12	0.206
UL-EP	−0.006	0.946	−0.001	0.988
LL-EP	−0.131	0.170	0.115	0.226
Z-Angle	0.017	0.858	−0.092	0.335
FH-N’Pog’	−0.127	0.182	0.011	0.909
N’-SN-Pog’	−0.01	0.916	−0.09	0.347

* indicates *p* < 0.05; ** indicates *p* < 0.01.

**Table 4:** Correlation analysis between cephalometric parameters and AHI in male and female subjects.

	Male		Female	
	r	*p*	r	*p*
SNA	−0.12	0.335	−0.192	0.205
SNB	−0.193	0.117	−0.325	0.029 *
ANB	0.168	0.173	0.265	0.078
Ptm-A	0.097	0.436	−0.152	0.318
Ptm-S	−0.052	0.677	−0.047	0.760
PP-FH	0.062	0.616	0.259	0.086
PP-GoGn	−0.044	0.726	−0.125	0.412
OP-SN	0.068	0.585	0.007	0.963
Go-Pog	−0.13	0.296	−0.222	0.144
Go-Co	0.028	0.823	−0.115	0.453
Pcd-S	0.089	0.475	0.026	0.866
MP-SN	0.065	0.604	−0.007	0.965
FH-MP	0.024	0.846	−0.028	0.857
SGn-FH	0.08	0.522	0.163	0.286
NBa-PtGn	−0.127	0.307	−0.186	0.221
N-ANS	0.109	0.379	−0.01	0.946
ANS-Me	0.001	0.990	−0.296	0.048 *
S-Go	0.018	0.886	−0.195	0.200
S-Go/N-Me	−0.019	0.879	0.027	0.859
ANS-Me/N-Me	−0.065	0.598	−0.256	0.090
U1-L1	0.25	0.041 *	0.149	0.330
U1-SN	−0.284	0.020 *	−0.132	0.386
U1-NA (mm)	−0.134	0.278	−0.112	0.463
U1-NA (°)	−0.185	0.135	−0.029	0.848
L1-NB (mm)	−0.079	0.524	−0.061	0.689
L1-NB (°)	−0.199	0.107	−0.311	0.037 *
L1-FH	0.045	0.721	0.075	0.623
U1-APo	−0.003	0.979	0.01	0.950
L1-APo	−0.105	0.399	−0.324	0.030 *
U6-Ptm	−0.022	0.858	−0.238	0.115
U1-PP	0.02	0.875	−0.187	0.220
U6-PP	0.122	0.324	−0.19	0.211
L1-MP	0.114	0.360	−0.189	0.214
L6-MP	0.102	0.409	−0.236	0.119
UL-EP	0.187	0.129	−0.055	0.720
LL-EP	−0.037	0.766	−0.105	0.492
Z-Angle	−0.029	0.817	−0.095	0.534
FH-N’Pog’	−0.071	0.567	−0.2	0.188
N’-SN-Pog’	0.036	0.772	0.112	0.465

* indicates *p* < 0.05; ** indicates *p* < 0.01.

**Table 5:** Correlation analysis between cephalometric parameters and LSaO_2_ in male and female subjects.

	Male		Female	
	r	*p*	r	*p*
SNA	0.075	0.548	0.134	0.381
SNB	0.008	0.951	0.36	0.015 *
ANB	0.036	0.775	−0.319	0.033 *
Ptm-A	−0.092	0.458	0.235	0.120
Ptm-S	−0.111	0.371	−0.11	0.470
PP-FH	0.071	0.570	−0.137	0.370
PP-GoGn	0.064	0.608	−0.091	0.551
OP-SN	0.129	0.299	−0.056	0.717
Go-Pog	−0.089	0.473	0.274	0.069
Go-Co	−0.229	0.062	0.174	0.252
Pcd-S	0.021	0.865	0.037	0.811
MP-SN	−0.009	0.945	−0.119	0.437
FH-MP	0.067	0.587	−0.099	0.519
SGn-FH	0.128	0.301	−0.262	0.082
NBa-PtGn	0.013	0.918	0.341	0.022 *
N-ANS	−0.161	0.193	0.054	0.727
ANS-Me	−0.186	0.131	0.191	0.210
S-Go	−0.183	0.139	0.23	0.128
S-Go/N-Me	−0.002	0.988	0.067	0.661
ANS-Me/N-Me	−0.111	0.371	0.13	0.394
U1-L1	−0.14	0.257	−0.072	0.637
U1-SN	0.015	0.903	0.247	0.102
U1-NA (mm)	−0.064	0.609	0.167	0.273
U1-NA (°)	−0.053	0.670	0.208	0.171
L1-NB (mm)	0.087	0.483	−0.098	0.524
L1-NB (°)	0.136	0.274	0.098	0.521
L1-FH	−0.146	0.238	0.125	0.413
U1-APo	0.033	0.792	−0.101	0.508
L1-APo	−0.005	0.969	0.097	0.525
U6-Ptm	−0.18	0.144	0.325	0.030 *
U1-PP	−0.077	0.538	0.065	0.670
U6-PP	−0.276	0.024 *	0.148	0.332
L1-MP	−0.184	0.137	0.029	0.852
L6-MP	−0.178	0.150	0.103	0.501
UL-EP	−0.004	0.977	−0.173	0.256
LL-EP	0.054	0.662	0.079	0.604
Z-Angle	−0.157	0.204	0.099	0.518
FH-N’Pog’	−0.166	0.179	0.268	0.075
N’-SN-Pog’	−0.3	0.014 *	0.229	0.130

* indicates *p* < 0.05.

**Table 6:** Multiple regression for cephalometric variables associated with AHI in male and female individuals.

Gender	Variables	B	t	*p*	VIF	R^2^	F
Male	L1-NB (°)	−0.745	−2.407	0.019	1.395	0.124	2.984 *
	U1-NA (°)	−0.337	−1.462	0.149	1.017		
	UL-EP	1.025	1.392	0.169	1.390		
Female	SNB	−1.028	−1.568	0.125	1.684	0.218	2.795 *
	ANS-Me/N-Me	−1.644	−1.902	0.064	1.404		
	L1-NB (°)	−0.141	−0.359	0.722	1.137		
	U6-Ptm	0.327	0.376	0.709	1.929		

* indicates *p* < 0.05.

**Table 7:** Multiple regression for cephalometric variables associated with LSaO_2_ in male and female individuals.

Gender	Variables	B	t	*p*	VIF	R^2^	F
	U6-PP	−1.119	−1.831	0.072	4.588	0.202	3.084 *
	Go-Co	−0.049	−0.237	0.813	1.766		
Male	ANS-Me	0.989	2.837	0.006	7.038		
	L1-MP	−0.932	−2.494	0.015	3		
	N’-Sn-Pog’	−0.526	−2.894	0.005	2.037		
	SNB	0.231	0.694	0.492	3.958	0.469	6.881 **
	ANB	−1.361	−3.608	0.001	2.205		
Female	Go-Pog	−0.022	−0.123	0.903	3.159		
	U6-Ptm	0.59	1.998	0.053	2.022		
	SGn-FH	0.471	1.791	0.081	2.958		

* indicates *p* < 0.05, ** indicates *p* < 0.01.

## Data Availability

The original contributions presented in the study are included in the article; further inquiries can be directed to the corresponding authors.
